# Centring the voices of survivors of child sexual abuse in research: an act of hermeneutic justice

**DOI:** 10.3389/fpsyg.2023.1178141

**Published:** 2023-12-06

**Authors:** Susanna Alyce, Danny Taggart, Angela Sweeney

**Affiliations:** ^1^School of Health and Social Care, University of Essex, Colchester, United Kingdom; ^2^King’s College London, London, United Kingdom

**Keywords:** hermeneutic injustice, testimonial injustice, child sexual abuse, mad studies, participatory research, lived experience, trust

## Abstract

Survivors of child sexual abuse (CSA) are known to hold silence and create distance between themselves and service providers for self-protection, as groomed behaviour or to protect the listener from vicarious trauma. Silence for many survivors has also been reinforced as a beneficial action by previous experiences of disclosing and being rejected, challenged, or disbelieved. How can researchers be sure the same dynamic is not playing out in research interviews? Generating reliable research data is an imperative and an act of epistemic justice that enables CSA survivors to testify to the suffering caused by abuse and subsequent trauma distress and to contribute to social discourse for change. Fricker, however, notes that the precursor to testimonial justice is hermeneutic justice. Hermeneutic justice pivots on the dual action of accurate understanding and interpretation, but CSA experiences may be beyond the comprehension of untraumatised listeners because their own frame of reference renders them unable or unwilling (even if unconsciously) to entertain the truth of such human depravity and cruelty. If survivors are not understood, their testimonies can be misconstrued or oftentimes excluded from the generation of epistemic knowledge, leaving the survivors unable to make sense of, and process, their experiences. These are crucial issues for researchers in the field of CSA and other crimes of sexual and gendered abuse. This study considers the operationalisation of a participatory research approach held within a lived experience research paradigm. Such methodologies advocate for peer involvement, which is becoming more widely recognised as supporting testimonial justice and the accurate understanding and interpretation of survivors’ testimonies. The issue of validating the methodology and methods is considered, exploring a rigorous data audit and researcher reflexivity as contributors to trustworthy data. Peer and participant safety when researching through lived experience is addressed. Data from a doctoral research study are used to illustrate this article.

## 1 Introduction

Child sexual abuse (CSA) and the traumatic distress that victim–survivors live with may be beyond rational comprehension (Herman, 1992; Freyd, 1996; van der Kolk, 2014), but parts of society now seem ready to listen, as the final report of the Independent Inquiry into CSA has shown (IICSA, 2022). However, what action will emerge in the wake of the large-scale listening exercise at IICSA remains to be seen. Recently, there have been calls for detailed and focused research in the fields of gendered and sexual violence (James Lind Alliance, 2022). However, researchers need to be aware of how their own role in the research process may be shaping outcomes (Sweeney et al., 2009).

The need for robust data encounters at least one well-recognised barrier: the silence that CSA survivors hold around disclosure. Survivors have described experiencing misunderstanding, challenge, and rejection when disclosing the events of their childhood and CSA’s trauma imprint of distress (Alaggia et al., 2019). These experiences reinforce the utility of silence and may have arisen from epistemic injustice, when what happened, and is happening, falls into “the gap in collective interpretative resources [which] puts someone at an unfair disadvantage when it comes to making sense of their social experiences” (Fricker, 2007, p. 1). Fricker is talking of hermeneutic injustice, the precursor to testimonial injustice. These two elements comprise epistemic injustice. The words of one participant in a recent qualitative study of CSA illustrate exactly this: “I wish people could be in my skin for a day and just understand” (Tessa, CSA survivor and research participant).

Understanding is a crucial issue when conducting research studies with CSA survivors, and adaptive methodologies addressing this may be transferable to research studies investigating other forms of sexual and gendered violence. One approach is to work within participatory research paradigms. These include ethnographic (LeCompte and Schensul, 2010) and autoethnographic (Jones et al., 2016) methodologies, community-based participatory research (Minkler and Wallerstein, 2003), participatory action research (PAR) (MacDonald, 2012), and peer research (Bizieska and Johnston, 2015). Key to participatory research approaches is a blurring of the boundaries between researchers and participants, where people with lived experience become co-researchers at all stages of the research. Participatory studies often have a commitment to tackling marginalisation and exclusion. However, there remains a separation between researchers and participants because researchers in participatory paradigms tend not to have – or not to disclose that they have – lived experience.

This is where survivor research differs. Within survivor research, there is also a commitment to co-research with participants and to address exclusion, but the main researchers share an identity with research participants (Sweeney et al., 2009). Russo, therefore, described survivor research as the most extended form of participatory research, commenting that it “values first-person experience which it considers a true and legitimate source of evidence” (Russo, 2012). Similarly, Mad Studies describes a body of mad-positive knowledge that places first-person experience as central to our understanding of phenomena (Beresford and Russo, 2022). Thus, the unifying feature of survivor research and Mad Studies is the value placed on experiential knowledge as both an adjunct and a challenge to clinical and academic epistemology.

A recent qualitative study used a participatory approach to explore survivors’ experiences of trust and trustworthiness. It was designed to not only address issues of epistemic injustice through privileging survivor accounts but also using a survivor research paradigm (Sweeney et al., 2009; Faulkner, 2017). This meant that survivors’ experiences were more likely to be understood by the researcher due to a shared epistemic frame around CSA. (Re)building trust between CSA survivors and people in positions of authority is crucial in the provision of services, including but not limited to therapeutic or clinical practice (Parry and Simpson, 2016). However, to generate data that speaks to this need, participants needed to trust the researcher. Since the relationship between researcher and participant is short lived and yet designed to elicit sensitive and potentially shameful narratives, this presents an awkward problem. The study design addressed this central issue of survivor–participants’ previous experiences of hermeneutic injustice, and this article presents and discusses how the study’s participatory approach was a facilitator of testimonial justice. The study design centred the primary researcher’s shared experience of CSA to overcome issues of shame and other reasons for participants holding silence, to flatten power hierarchies and to offer safety and agency to participants. This study considers the central issue of understanding and interpreting CSA data empathically and accurately as hermeneutic justice in action. Verification of the study findings as trustworthy, using a robust data trail audit and researcher reflexivity, are discussed. Finally, issues of researcher and participant safety and well-being are considered. This article focuses on evidence from the study that speaks specifically to epistemic and hermeneutic justice issues, while findings from the study regarding trust and trustworthiness in service provision are forthcoming.

## 2 Study design: key issues

### 2.1 The survivor of CSA

It is well documented that survivors of CSA hold silence around the abuse they have suffered because of groomed expectations of the negative effects of speaking out for themselves and their families (McElvaney, 2015). In the current study, Stella said: “There was a long period of time when I did not share any information [concerning CSA] with anyone and I was 37 before I ever shared anything with anyone.” Additional contributing factors reinforcing silence include fragmented memories caused by trauma (Sinason and Conway, 2022) and a sense of shame (MacGinley et al., 2019). Many survivors who have attempted to seek help at earlier stages in their lives speak of encountering rejection, blame, challenge, and disbelief (McElvaney, 2015; Rouf et al., 2016; Alaggia et al., 2019), and this can result in withdrawal. Chloe tried to disclose to her family GP as a teenager, but his questioning had this result: “I did not feel like the trust was there so I just closed down and left and walked out.” Chloe did not seek help again until her 30s. Additionally, survivors in this study spoke of their wish to protect others from the harmful effects of vicarious trauma until they were sure the listener was sufficiently resilient to hear narratives of abuse and trauma distress. Patrick said: “At first you are very cautious because first, what you are going to tell this woman is going to blow her mind.”

Survivors know that it can be difficult for non-survivors to understand the complexity and nuance of their trauma-related distress. Jake said: “They [clinicians] do not understand, it’s sometimes, it’s the tiniest, littlest sort of subtle things that are the most painful, I was sexually abused for 6 years but it was that moment when my dad [non-abusing parent] did not trust me that was hardest.” The experience of not being understood was foregrounded in reports by IICSA and the Truth Project (IICSA, 2022; IICSA: Truth Project, 2022). While these experiences arose within relationships of service provision, they may equally arise in the researcher–participant relationship involving abuse narratives and create dilemmas when shaping research studies.

### 2.2 Testimonial justice

Experiences of seeking help and then having disclosures challenged or rejected can arguably be considered a form of testimonial injustice. In this study, Jake said: “I tell you, I tried to disclose to teachers, um who kind of brushed it under the carpet, told me I was making too much of it. […] I do not think many people did not believe me, they just dismissed the experience and that I was using it as an excuse to be lazy.” The GP that Chloe disclosed to said: “Are you sure, could you have [misconstrued] this?”

Scholarship-advancing theories of epistemic injustice, particularly in oppressed and marginalised populations (Dotson, 2014; Pohlhaus, 2020), posit that testimonial injustice rests on the listener failing to vest credibility in the speaker (McKinnon, 2016). One reason for this is that when a speaker voices a social experience that is beyond the comprehension of the listener, it renders the experience incomprehensible (Falbo, 2022). This then robs the speaker of the chance to make sense of their experiences, leaving them marginalized and excluded from discourse.

Survivors often wait many years before disclosing, and disclosures usually emerge piecemeal as trust is built with the recipient (Alaggia et al., 2019). Additionally, trauma memories are known to be fragmented and may not present as a chronologically smooth timeline (Sinason and Conway, 2022). This can make listening difficult for some recipients because it lies outside their frame of reference, and they cannot conceptualise what they are hearing. This can happen because of a lack of culturally sanctioned narratives around CSA for the speaker and listener to draw on, and so, the interpersonal injustice between two people links to a wider social injustice. Thus, the listener needs to engage in “reflexive critical sensitivity” (Fricker, 2007, p. 7) and trust in the speaker’s testimony. The benefit of such virtuous engagement was evident in Yasmin’s description of how she came to realise that so many of her difficulties were emanating from her CSA experiences:

*“She [therapist] never lectured me or or or tried to dig in a way that was, that I got defensive, she was very listening and listening and listening and finally I decided, and also I told her I think there’s something wrong with me, I think something is really really really wrong with me, either I’m bi-polar or I’m a borderline person or something is wrong with me, something is majorly wrong with this, and she was like what is this, what is this, where does it come from and she was always asking me and this when I realised that maybe this [CSA] is what everything is about.”*

The sexual abuse of children is morally abhorrent, and yet it has been consistently difficult for modern societies to engage with (Rouf and Taggart, 2022), leading to pendulum swings between outrage and denial. Herman (1992) offered one explanation for this when she says of the wider issue of trauma: “The study of psychological trauma does not languish for lack of interest. Rather, the subject provokes such intense controversy that it periodically becomes anathema” (Herman, 1992, p. 7).

In another way, testimony can cause a recoiling from the evidence as it raises the possibility that the very fabric of society is ethically unsound (Herman, 1992; Fassin, 2009; van der Kolk, 2014). Recognising the scale of CSA creates a challenge to institutional structures that are “too big to fail”, and the injustice of denial of the survivor’s testimony is a small price to pay for the preservation of the status quo (IICSA, 2022). The interplay between institutional failures to believe victims and epistemic injustices in interpersonal contexts was explored in the work of the Truth Project (Barker et al., 2023).

### 2.3 The researcher’s role

These many and varied issues mean that providing CSA survivors with a safe context for sharing testimony is essential. Testimonies must be received by researchers with the virtuous ability to listen. The operationalisation of such a “virtuous ability” (Fricker, 2003) offers validation to the survivor–researcher as an epistemologically virtuous agent. Coady forwarded the concept of a “learning mechanism” (Coady, 1992, p. 47) that enables the listener to gradually establish the trustworthiness of the particular speaker over a series of interactions. In this way, the survivor–researcher builds “critical capacities” which are non-inferential and operate innately, meaning that while listening, the capacity to believe and understand is unreflective but not uncritical. It is the very fact of the researcher having a CSA history that is the “learning mechanism,” providing the ability to critically assess the survivor–participant’s testimony as a true representation. Within this are the seeds of accurately presenting testimony in research data. The next issue is to find a shared understanding of “accuracy” between the reader and researcher, and this depends on the hermeneutics of the study.

### 2.4 Hermeneutics: accurate and sensitive interpretation

Hermeneutics is, in essence, an interpretation that seeks to make the “unintelligible both intelligible and communicable” (Dyer, 2010). Watts (2014) considers the juncture between two elements, interpretation and understanding, in qualitative research. He, like many others, rejects the notion of value-free interpretation because of the inevitable subjectivity of researchers because they are human. Instead, he advocates for the importance of the researcher shifting her proximity between “closeness” when understanding participants’ words, and “distance” when conducting analysis using theoretically and methodologically informed viewpoints (Watts, 2014). To facilitate closeness, Ratcliffe’s phenomenological perspective may be useful. He theorises that understanding traumatised people and others with extreme psychological distress requires a “radical empathy.” This is a “way of engaging with others’ experiences that involves suspending the usual assumption that both parties share the same modal space” (Ratcliffe, 2012, p. 483). Distance, on the other hand, is facilitated by the more traditional skills of the academic researcher. Interpretative Phenomenological Analysis (IPA) lends itself to the operationalisation of the hermeneutics of CSA testimony, given its foregrounding of interpretation. IPA has tackled the otherwise obfuscated issue of “not enough,” “too much,” and “incorrect” interpretation head-on. This article is not the place to play out the debates around IPA (see Smith, 2011; Smith, 2018; Nizza et al., 2021). However, IPA does provide a theoretically well-explored paradigm for survivor research, in that the virtues extolled in epistemic justice have been acquired through shared CSA experience and actively inform the interpretation in the “close” and empathic way these various scholars are advocating (Ritunnano, 2022).

Given these precursors, research into CSA needs careful consideration of how to create an environment where participants feel safe enough to offer their testimony in approximately a 1 h interview. The interviewer/researcher has the virtuous sensibility to offer hermeneutic justice. Participatory approaches that centre on lived experience offer one solution to this predicament.

### 2.5 Lived experience methodology

Lived experience as valid epistemology challenges the more traditionally established and valued positivist and (supposed) objective study of those receiving care (Sedgwick, 1982; Beresford, 2021). Lived experience is central to participatory ideology and methodology, and is “knowledge that is generated from people with direct experience of the social issue under investigation” (Taggart, 2022, p. 155). Ethnographic approaches, well established in mainstream academia, address issues of social and cultural import (LeCompte and Schensul, 2010) and have long held such experience as valid epistemology. Ethnographic approaches have been bolstered by positioning researchers’ knowledge and sometimes shared identity through autoethnography (Jones et al., 2016). Recognising in this way the role the researcher’s life experience plays in shaping research has been foregrounded since the 1960s (Bruyn, 1966). Participatory Action Research (PAR) has advocated since the 1940s (Baum et al., 2006) for the generation of knowledge by, and the implementation of policy for, the people directly affected by the issue under research.

Thus, lived experience as an organising principle for research is in no way new. Lineages of oppressed people have claimed their right to self-research and self-identify and take their place in discourses concerning their histories. Colonial, feminist, queer, disability, and gender studies are established as respected epistemology. Mad Studies is now recognised within this umbrella (Beresford and Russo, 2022). “Mad” is not an acronym or abbreviation but a simple reclamation of the term by those living with mental distress. Mad Studies is both academic and an activism-oriented resistance to hegemonic systems of psychological care (Sweeney et al., 2016). Proponents include people suffering iatrogenic harm by psychological and psychiatric services *plus* those who identify as “mad positive” (Spandler and Poursanidou, 2019), meaning those who align themselves with the scope and mission of Mad Studies. Survivor research is both an ally to and a forebear of Mad Studies (Sweeney, 2016; Beresford, 2016b), and both share the focus on trauma-informed research, which a growing body of writing advocates for when working within mental health contexts (Sweeney et al., 2016; Shimmin et al., 2017; Edelman, 2023).

Electing to research a population of survivors of CSA is a statement of the use of the orienting trauma-informed lens. This approach asks, “what happened to you?” (Sweeney and Taggart, 2018), rather than using a diagnostic category or potentially pathologised grouping via symptoms. Trauma-informed care advocates for transparency, safety, intersectionality (Crenshaw, 2017), active listening (Rogers and Farson, 1957), empathy, and understanding (Elliott et al., 2005; Huang et al., 2014; Sweeney et al., 2018). These principles are more readily operationalisable when all parties understand their utility and provenance as being the opposite of the primary abuse that gave rise to trauma distress in participants within the research process (Rose, 2009; Survivors Voices, 2022).

This article will now look in more detail at how lived experience methodology was operationalised in one study of CSA survivors’ experiences of trust and trustworthiness.

## 3 Study design: operationalisation

### 3.1 Creating safety

The study design was informed firstly by the Charter for Engaging Survivors (Survivors Voices, 2023), produced by an abuse survivor-led charity, and secondly, by the guidance of an advisor who is a CSA survivor with more than 20 years of experience working with CSA survivors as a counsellor and trainer. Finally, ethics approval was obtained from the University of Essex (ref 18,014). The ethics application necessitated incorporated features to address the safety and support of the researcher and all participants and create an environment facilitating epistemic justice.

The Charter for Engaging Survivors calls for transparency as a counterbalance to the obfuscation and deceit of the original abuse and a flattening of power hierarchies in contrast to the abuse of power embedded in CSA. A key feature of this study was the explicit declaration of the CSA history of the researcher in every communication, which created a flat(ter) power hierarchy. This clear explication of identities speaks to openness and honesty and signalled to potential participants that their experiences would be understood and in no way stigmatised or demeaned. This was confirmed by Ruby, who said: “I feel it’s also easier for me to talk to you because you have experienced something, like, we have a level playing field.”

Participants were recruited using a “snowballing” method (Gilbert and Stoneman, 2016), whereby the researcher spoke to survivors already known to her and colleagues who worked with CSA survivors. This verbal invitation and explanation allowed for a personalised description of the research study, emphasising the importance and value of recruiting participants in a way that felt safe for all concerned. From these initial inquiries, survivors started contacting the researcher to ask for further information, which was given via email or personal communication. Initial contact was followed by emailing the participant information sheet and consent forms approved by the University of Essex Ethics Committee. Further recruitment was facilitated by a question at the end of each interview, asking participants to mention the study to survivor friends or colleagues and pass on the researcher’s details. This gave choice and agency to potential participants, who could make contact if they were interested in participating. This may appear to be a standard method for recruitment, but was essential in this study because it meant that the researcher was not an unknown and distant person, but someone known to the recommending link in the chain. This “word of mouth” recommendation helped survivors feel more at ease in knowing who they were speaking to when the interview began. As Chloe said: “The first time I met you [at University via introduction], I knew I wanted to help you […] there was just something about you, I knew I wanted to help you.”

The study was presented in all communications as an opportunity for “us” as survivors working together to gather and present data to inform service providers. This seemed to act as a motivator to participants, and their desire to be heard was evident. Yasmin said:

*“I’m grateful for being asked to participate in this so thank you for listening and thank you for sharing also your personal stuff and also for meeting me exactly where I am, and not, that’s also very big, to just being able to jump around things and being distant or this or that, just to blaaaaaah and babble on about it, so thank you.”*

Yasmin is making another important point about the impact of trauma on memory, which can come in bursts, oftentimes with an emotional charge and without chronology (Sinason and Conway, 2022). This may be important when considering the flow of an interview. The researcher herself (SA) has lived experience of the past bursting into the present, where narratives and memory do not come in a smooth, well-considered flow, which meant she could appreciate participants’ difficulties. Participants were understood and not demeaned for this, and this shared understanding lessened the need for, or expectation of, a narrative that started at the beginning and progressed through the middle to the ending.

Moments where emotions came to the fore were held sensitively. As the researcher (SA) understands the territory of abuse and trauma distress, such emotions did not scare her or give cause for undue concern. The material was not unfamiliar and so SA could tolerate the raw and sad experiences being recounted. It is possible that a different survivor–researcher might have struggled to receive the narratives, and it is recognised that every individual will have their own ideographic response in relationship to other survivors and when listening to testimony. Importantly, one can recognise that stigmatisation will almost certainly be avoided in conversations between CSA survivors.

Participants were pleased not to be closed down when emotions accompanied their narrative. Tessa said of her tears: “This is no worse than every day, it’s just I had to get it out and this happens when I go to therapy because it comes out, […] so it’s fine absolutely, I promise you.” Other studies investigating CSA placed power in the hands of the researcher to close the conversation if their participant became distressed (Banyard et al., 2001), thus robbing the survivor of her agency (Sen, 2019).

### 3.2 Survivor agency

The semi-structured interviews were conceived as “co-constructed” in the feminist model (Oakley, 2005), to negate, or at least work towards flattening, the power hierarchy of researcher (professional/expert) and participant (Jenkins, 2019). The interview schedule was shaped in discussion with the advisor, himself a survivor (see above), and a pilot interview with him was undertaken. Issues and options for flexibility and choice were explored and discussed during these foundational meetings. These choices again aimed at reducing power imbalances (Lyons and Chipperfield, 2000). The researcher attended interviewer training to refine her active listening skills (Cegala et al., 2000; Weger et al., 2014). The researcher and participant opened their time together with gentle, reassuring introductions to build rapport. The interviewer described her motivation to conduct the study, her history as a survivor, and her wish that their time together might be a chance for them to discuss issues around trust, rather than a question-and-answer session. The interview schedule was shared and discussed with each participant at the start of the interview to see the range of topics the researcher felt might be of interest. However, the participants could speak about whichever topic was most pertinent to them. In total, 17 participants were interviewed. Because the value of the lived experience was enshrined in the shape of each interview, participants were given choice and agency in the generation of data they felt relevant to the topic. This is another issue the Charter for Engaging Survivors highlights as a counterbalance to CSA, where choice and agency are negated (Survivors Voices, 2023). Above, Yasmin is expressing her gratitude for the possibility of sharing her lived experience with the researcher in the hope that it will inform professionals working with survivors of child sexual abuse.

Choice extended to the participant and researcher (SA) discussing and choosing the location for the interview. Some participants chose a café; others chose the university, their therapy centre, or an online video call. Concerns for the safety of the researcher when meeting unknown participants were built into her side of agreeing to a location. This was not the only or primary concern but was held in balance with the participants’ wishes.

These facets contributed to an environment of safety for the participant and the researcher. Tessa said: “I feel safe, I feel safe, I know you do this stuff and I know it’s happened to you, and you have just got a nice vibe to me so, you know, so it’s fine.”

Perhaps the environment of safety contributed to a relationality characterised by trust between participants and researcher, which facilitated the sharing of detailed and sensitive data. As Chloe said: “If I trust you you’ll get it all out of me, so obviously I must trust you.” Tessa echoed this: “I do not expect you to do anything terrible, (laughter), I do not think you have got a hidden agenda.” Tessa is pointing to this particular researcher (SA) having what Fricker (2003, p. 157) describes as a “sensibility” as an aspect of the “inferential model,” where testimony is being believed with “critical openness.” In this model, the listener does not simply listen with credulity to testimony, but has the developed virtue to be able to assess for truth while listening. Tessa knows her testimony will not be used against her materially or in any sort of shaming capacity. This is demonstrative of the survivor–researcher’s capacity, and ability, to operationalise both testimonial and hermeneutic justice because of the necessary virtues developed directly as a result of her own history of CSA. This both evidences Fricker’s theory and endorses Mad Studies as, at least, a suitable approach to research the sensitive and emotionally charged subject of sexual abuse.

Facilitating honest narratives addresses issues of testimonial justice, and the participants in this study were generous with the data they shared regarding their experiences of trust and trustworthiness. However, facilitating testimonial justice is only half of the dynamic underpinning epistemic justice: the other half is hermeneutic injustice, as delineated above. How can the researcher be sure that she is understanding her participants and interpreting their words accurately before going on to represent them in a framework of meaning that other readers can access?

### 3.3 Understanding

Finding a service provider or therapist willing, or able, to do this had been difficult for many participants: “You may never find that right person, you can probably go for years and years and years, go to different counsellors, different people and never find that right connection,” as Chloe said. Yet, the need to be understood was clear in the interviews, as expressed by Tessa above: “I wish people could be in my skin for a day and just understand,” and Helen: “I want understanding, of like why I’m ticking like the way I tick.”

Finding someone who has the capacity to understand had proven difficult for many of the participants in this study. Chloe said:

*“Do not tell me you [speaking of a doctor] understand, yeah, because you do not, you might have sympathy you have empathy, a lot of people have empathy over it and they think they know how it feels and ‘cos once they find out they feel uncomfortable, […] yeah, and then they have that sort of feeling of uncomfort [sic] but you do not actually understand.”*

Chloe, now working with survivors as a mental health nurse, said: “…and there’s just no understanding, you can just see they [health professionals] do not understand any of it, I’ve sat in numerous reviews and I’ve said my piece as well and straight away they have [given a diagnosis], you are labelling someone with the wrong label and you know I’ve worked with people, young people who have been sexually abused […] and that they do not ever, I found they did not bring that into it … they [the doctors] sort of brushed over it… because they do not understand it.” It can be argued that such doctors “brush over it” because the genesis of the issues, CSA, is not important in a biomedical formulation of the patient’s mental health. Many, including the anti-psychiatry and the Mad movement, argue against that and instead suggest that such a view might be indicative of a history of denial (Beresford, 2016a). However, once it is recognised that the trauma of CSA is a harm in its own right, it no longer matters whether it fits the epistemological formulation or not: it warrants recognition on an ethical basis. The ethical listener would perceive the “moral colouration” (Fricker, 2003, p. 160) of the issue, irrespective of their worldview, but here they have lacked the “ethical socialisation” (p. 160) of seeing CSA as a central organising feature of survivor experience. This compares unfavourably with the survivor-centred approach, which arises from the survivor–researcher’s socialisation by way of direct experience.

This is echoed by Jake, who is a CSA survivor–educator and therapist. He said: “I was training some psychologists and they said what model of recovery do you use, and I simply said I just ask people what they need and what help they’d like, and they said that’s so radical and so amazing, I said no, I was thinking, no, it’s just being a human being […] it’s about humanity.”

The recognition of the value of speaking with someone who has been through CSA is signalled in the earlier quotes and also by Betty, who said: “You’ve [the researcher] really gone there and you know, really looked into it and been absolutely honest and brutally honest and […] I thought you have been through all this […] so it sort of made me feel absolutely safe to tell you because you would get it.”

Survivors in this study spoke repeatedly about a process of healing unfolding as a result of a listener understanding and that this enabled trust to flourish. They also said that understanding is crucial because it brings mutuality, which in turn symbolises a shared humanity, and through this, the self-worth of the survivor was affirmed. Betty discovered she could trust her GP and shared more of her history of CSA with her: “So she [GP] said, “Oh you have done really well” and somehow her saying it just made it feel like “Oh my god yeah”, I had not realised that I’ve survived it and I’ve done OK you know […] she said it and it made it real.”

This example directly illustrates how understanding by the listener results in a shift in self-conceptualisation, as Fricker posits: “A virtuous hearer may effectively be able to generate a more inclusive hermeneutic micro-climate through the appropriate kind of dialogue with the speaker” (Fricker, 2007, p. 171).

### 3.4 Interpretation

Understanding is the first component of hermeneutic justice, according to Dyer’s definition given above, and interpretation is the second. In a participatory paradigm, it is imperative that a person of lived experience is offering the interpretation because of the power of the “double hermeneutic.” This term emerged from the philosophy of phenomenology and denotes the recognition that in every living moment, a human being is making meaning from the information arriving in their consciousness, and a researcher is then making meaning of the meaning their participant has made (Eatough and Smith, 2017). This circles back to the value of someone with the radical empathy of lived experience accurately interpreting the words of the participant, and these two levels of interpretation are transparently presented in the study findings for the reader to have the opportunity to assess the validity of the data. This is more than an echo of the advocation for virtuous sensibility rendered above. Recognising the double hermeneutic gives the reader the opportunity to notice their own meaning-making process as they read, and this is a third hermeneutic level (Smith et al., 2009).

To match IPA’s requirement for explicit interpretations, the study used to illustrate this article included both lengthy participant quotes alongside the interpretative argument from the researcher. This is called for in all IPA studies (Nizza et al., 2021). However, this study also included a 5,000-word appendix with further substantiating participant quotes footnoted in the findings chapters. In this way, the survivor’s voice was evident in the study findings. Furthermore, the appendix and extensive quotes were intended as a mark of respect for the participants’ generosity in sharing their narratives, which many times were raw and shaped by iatrogenic harm. This inclusion of extensive quotes meets the need for those wishing to audit the study as valid, provides a data trail from transcripts through to conclusions, and is in line with the JBI Checklist for Qualitative Research (JBI, 2017).

An example from the study may illustrate hermeneutics in operation:

“Listening included embodied engagement:

*“how can you trust how can you trust someone who do not look in your face” [Helen].*

And when listening, a trustworthy other is not preparing their response. Listening is not just to use the survivor’s words to springboard into their opinion or view. Staying with the survivor in their narrative was important. For Chloe, her trusted therapist:

“*did not try to put their two pence in all the time” [Chloe].*

This metaphor suggests that Chloe appreciates her words being valued and the trustee not valuing their own words more than hers. This valuing appears in Jo’s statement too:

*“listening um, about taking seriously what someone else is saying not only thinking about your own self and your own response but really listening to the other person” [Jo].*

*“just feeling that somebody is listening actually, taking it in and not just filing it away” [Anna].*

Not “filing it away” suggests the listening is engaged, and the words are not being dismissed, as in filed away, but also not being added to her medical notes or files. Her words are valued as live and relevant, not as indicators of disorder. In this way, these survivors found listening indicative of being given worth.”

This excerpt reflects the researcher’s (SA) endeavour to discover what meaning the participants were making of the capacity of service providers to listen and understand them before deciding if the person was trustworthy and thus able to be endowed with further CSA details. The meaning the researcher is making is the second hermeneutic in action – using metaphor (“filing away”) as an insightful indicator of meaning. This section also reinforces the epistemic virtue of listening that contributes to the reversal of the marginalisation of groups who are not understood or epistemically valued. This avoids Fricker’s “hermeneutical hotspots – locations in social life where the powerful have no interest in achieving a proper interpretation, perhaps indeed where they have a positive interest in sustaining the extant misinterpretation” (Fricker, 2007, p. 152).

### 3.5 Reflexivity in operation

Guidance on generating robust qualitative research often expounds on the need for reflexivity in the study design and process (Finlay et al., 2003; Mann, 2016; Dean, 2017). Most PhD studies require a statement of the researcher’s stance and engagement with reflexivity. In this study, reflexivity proved essential as a tool for accurate data generation, but also to support the researcher. A reflexive journal was maintained, both written and in audio recordings, and the transcripts were notated with reflexive commentary as the iterative process of repeated reading unfolded. Towards the end of the study write-up, an autoethnographic chapter was written as an exercise in overviewing the process and the researcher’s part in shaping the findings, discussion, and conclusion. This excerpt illustrates how reflexivity informed choices about data collection and inclusion:

### Transcript three: Susanna

*“After the pilot and first interview I found I was uncertain about the amount of time my own voice was taking up of the 1 h allotted. I realised that I had a lot to say of my own experiences of trust and trusting, and this was stealing valuable airtime from my participants. I reflected that I could perhaps gather this information a different way, because while I wanted to have myself in the research in a way that authentically represented my journey as a survivor, I also needed a reflexive practice to see where my experience aligned with, or differed from that of the participants. This gave rise to the interview conducted with me by my person-centred therapist, using my own interview schedule. This recording remained unlistened to until after the first draft of the findings. I decided not to include my words as data in order to keep my distance from the findings, but instead I have used it to reflexively critique when (and whether) my views have shaped my interpretations of individual moments within transcripts and the amalgamating of data into final chapters.”*

*“When I did listen to myself speaking, shocked is not too strong a word for what I felt. Almost everything I said echoed and mirrored the findings, but at the time I recorded it I had no notion that my experiences fitted the shape of the process flow chart, or the relationship between generalised and relational trust. I had no idea that my rough-hewn definition of trust would match the other participants’ personal construct of trust (see conclusion). I remember during the interview feeling that I was rambling around the subject, tangentially answering the questions, and said at the end that I feared I had not been able to give any valuable data and did not have a clear overview of my own trust abilities or experiences. Even this statement was echoed by Anna at the end of her interview!”*

Working with narratives of trauma can be triggering (Sweeney and Taggart, 2018; Alyce, 2022a), and reflexivity enables a “stepping back” to check in with the unfolding of vicarious or triggered reactions to the work. Having a supervisor (DT) with lived experience was helpful and important in giving an overview of the way the study moved between domains of personal and professional (McWade, 2020). Furthermore, it created a context of ethical socialisation to the topic, and the hermeneutic spaces that were established together had epistemic justice as a central task. In this way, the research was about discovery but also epistemic support for survivor accounts, and as such, moved beyond issues of credibility to deeper concerns around dignity and worth. The researcher (SA) gained further reflexive support from an online survivor-researcher peer-support group run by Survivors Voices, authors of the Charter for Engaging Survivors (see above).

The role of survivor–researcher was helpful in the ways this article has illustrated, but it also brought challenges. During the first COVID lockdown in 2020, the researcher (SA) became physically unwell due to the stress of COVID-19, shingles, and perhaps spending uninterrupted hours transcribing and analysing transcripts. She was helped through this by her supervisor, giving her extra time to complete the work and with personal counselling. She scheduled additional time for self-care, using exercise and meditation as a grounding tool. By recognising the dual role of reflexivity as a method of generating trustworthy research and offering the capacity to protect and support, more time engaged in reflective practice became essential to carry the study to its completion (Alyce, 2022b; Alyce, 2023).

## 4 Conclusion

This article has presented an argument for good practice in the study of survivors of CSA by employing the lived experience of a survivor-researcher working within participatory paradigms and points to the necessity to support this approach with robust supervision and personal counselling. This modality has the ability to rebalance power hierarchies, create safety, allow agency and understanding for participants, and effect accurate interpretations. The robust implementation of this necessitates a well-documented data trail for audit and reflexivity to ensure the relevant proximity is adopted, while engaging with participants in person and analysis. This article has argued that when these elements are synthesised, Fricker’s thesis of virtuous sensibilities facilitating epistemic justice through the avoidance of testimonial and hermeneutic injustice is supported. Future research is recommended to replicate the approach with other traumatised populations, such as domestic violence and sexual violence. The article adds to the literature on conducting trauma-informed research (Edelman, 2023) and provides a framework that can support survivor–researchers and participants to engage in this hard but critical work in ways that glean data otherwise lost in paradigms where shame prohibits the speaking of participant truths.

## Data availability statement

The data analyzed in this study is subject to the following licenses/restrictions: unpublished research subject to PhD submission. Requests to access these datasets should be directed to DT, University of Essex, dtaggart@essex.ac.uk.

## Ethics statement

The studies involving humans were approved by School of Health and Social Care, University of Essex. The studies were conducted in accordance with the local legislation and institutional requirements. Written informed consent for participation in this study was provided by the participants’ legal guardians/next of kin.

## Author contributions

All authors listed have made a substantial, direct, and intellectual contribution to the work and approved it for publication.
